# Supplementary material for: The genome sequence of the Brindled White-spot moth, Parectropis similaria (Hufnagel, 1767)
Source: Wellcome Open Res. 2025 Apr 8;10:179. [Version 1] doi: 10.12688/wellcomeopenres.23984.1 (PMC12485357; doi:10.12688/wellcomeopenres.23984.1)
Supplement: Peer review history

# Reviewer report: Reviewer response for version 1

**DOI:** 10.21956/wellcomeopenres.26460.r122080

**Published:** 2025-05-08

**Authors:** Arjen Van 't Hof

**Affiliations:** 1 Biology Centre of the Czech Academy of Sciences, Budějovice, Czech Republic

The genome sequence of the Brindled White-spot moth has been added to the ever-growing list of genome assemblies produced by the Darwin Tree of Life Consortium. It is always enjoyable to read the background of the species before diving into the assembly details. In this case, the background is rather short, but this is not an oversight of the authors. The Brindled White-spot moth has not been studied in great detail, and therefore, there are not that many noteworthy facts to report.

 The manuscript follows the structure we are used to from Darwin Tree of Life genome assembly papers, except that there is more emphasis on species confirmation by barcoding which is now a standardized procedure. It is reassuring though that the initial species identification was done by the first author based on entomological knowledge. The assembly statistics are outstanding, in line with what we have come to expect from Darwin Tree of Life.

 The language is rich with jargon. I am still not entirely sure what ‘localised homologous

 pairs’ means to name an example.

 Figure 1 shows tubes coded FD20710430 and FD20710431, but these codes are not explained further in the figure legend or the main text, nor are these codes available in NCBI or EBI databases. It is not clear what these tubes are, what they contain, why there are two of them, and why they are in the photograph.

Are sufficient details of methods and materials provided to allow replication by others?

Partly

Is the rationale for creating the dataset(s) clearly described?

Yes

Are the datasets clearly presented in a useable and accessible format?

Yes

Are the protocols appropriate and is the work technically sound?

Yes

Reviewer Expertise:

Lepidoptera genetics

I confirm that I have read this submission and believe that I have an appropriate level of expertise to confirm that it is of an acceptable scientific standard.

# Reviewer report: Reviewer response for version 1

**DOI:** 10.21956/wellcomeopenres.26460.r122082

**Published:** 2025-05-07

**Authors:** Panagiotis Ioannidis

**Affiliations:** 1 Foundation for Research & Technology - Hellas, Crete, Greece

This paper describes the genome sequencing and assembly for *Parectropis similaria*, a lepidopteran insect. The assembly quality looks great and the data are publicly available for anyone who is interested in downloading and using them.

 I only have a minor comment; In the "Assembly quality metrics" section the authors say "...identified 98.5% of the expected gene set (single = 98.1%, duplicated = 0.5%).". I'd suggest that they change this to "...identified 98.5% of the BUSCOs...", since a "gene set" is a different thing.

 Looking at the deposited sequencing data, I noticed that there also exist RNAseq data. Were these used? For example, for gene annotation? Maybe the authors should mention this in the manuscript, because it is an important resource and it shouldn't go unnoticed!

Are sufficient details of methods and materials provided to allow replication by others?

Yes

Is the rationale for creating the dataset(s) clearly described?

Yes

Are the datasets clearly presented in a useable and accessible format?

Yes

Are the protocols appropriate and is the work technically sound?

Yes

Reviewer Expertise:

insect genomics, bioinformatics

I confirm that I have read this submission and believe that I have an appropriate level of expertise to confirm that it is of an acceptable scientific standard.

# Reviewer report: Reviewer response for version 1

**DOI:** 10.21956/wellcomeopenres.26460.r122079

**Published:** 2025-05-07

**Authors:** Annabel Whibley

**Affiliations:** 1 The University of Auckland, Auckland, New Zealand

Boyes and colleagues present the high quality reference genome assembly of the Brindled White-spot moth, *Parectropis similaria.  *The natural history, specimen collection, sequencing and bioinformatics workflows are comprehensively and accurately described, with excellent reporting of metadata using DToL templates. Public accession links are active. RNAseq data was obtained from the same individual used for HiC library construction but an assembly has not been provided at this time. The presented genome assembly is of high quality, achieving 7.C.Q65, with the failure to recover the W chromosome indicating that the karyotype is likely ZO. This is the first Data Note that I have reviewed where the manual curation protocol links are active (as opposed to  "in prep") and its is great to see this information now available.

Are sufficient details of methods and materials provided to allow replication by others?

Yes

Is the rationale for creating the dataset(s) clearly described?

Yes

Are the datasets clearly presented in a useable and accessible format?

Yes

Are the protocols appropriate and is the work technically sound?

Yes

Reviewer Expertise:

Genomics, Bioinformatics

I confirm that I have read this submission and believe that I have an appropriate level of expertise to confirm that it is of an acceptable scientific standard.

# Reviewer report: Reviewer response for version 1

**DOI:** 10.21956/wellcomeopenres.26460.r122078

**Published:** 2025-04-30

**Authors:** Laurence Despres

**Affiliations:** 1 Laboratoire d'Ecologie Alpine (Ringgold ID: 56837), Grenoble, Auvergne-Rhône-Alpes, France

The authors report on the chromosome-level genome assembly of an individual female specimen of the Brindled White-spot moth *Parectropis similaria *(Geometridae). The initial identification by morphology of the two collected specimens was verified by an additional DNA barcoding process.

 The genome sequence is 574.79 megabases in span. Most of the assembly is scaffolded into 31 chromosomal pseudomolecules, including the Z sex chromosome. The Z chromosome was assigned by synteny to *Agriopis aurantiaria *(GCA_914767915.1). This species appears to exhibit the ZO karyotype. The assembly was performed using appropriate methods : Pac Bio HiFi long reads (42-fold coverage) and Hi-C data to refine scaffolding, manual curation of the final assembly, and contamination check, following the analysis pipeline and high standard of the Darwin Tree of Life Project for results presentation.

 The mitogenome was also assembled. The quality of the assembly was further assessed by the proportion of complete BUSCO genes recovered, which is very high (>98% of Lepidoptera database BUSCO genes lepidoptera_odb10 set). Gene annotation was not performed, although RNAseq data was produced and made available. Protein-coding predictions would be a useful addition to this high quality genome.

 This high quality genome assembly will provide a reference genome for further genomic studies on this geometrid moth.

Are sufficient details of methods and materials provided to allow replication by others?

Yes

Is the rationale for creating the dataset(s) clearly described?

Yes

Are the datasets clearly presented in a useable and accessible format?

Yes

Are the protocols appropriate and is the work technically sound?

Yes

Reviewer Expertise:

population genomics

I confirm that I have read this submission and believe that I have an appropriate level of expertise to confirm that it is of an acceptable scientific standard.
